# The relationship between medical marijuana use and prescription pain reliever use among U.S. adults: A retrospective analysis utilizing the 2015–2019 National Survey on Drug Use and Health (NSDUH)

**DOI:** 10.1016/j.rcsop.2023.100368

**Published:** 2023-11-11

**Authors:** Tyler J. Dunn, Erin Holmes, Yi Yang, John P. Bentley, Saim Kashmiri, Sujith Ramachandran

**Affiliations:** a Department of Pharmacy Administration, University of Mississippi School of Pharmacy, University, MS 38677, USA; b Department of Marketing, University of Mississippi School of Business, University, MS 38677, USA

**Keywords:** Marijuana, Cannabis, Medical marijuana, Prescription pain relievers, Opioids, NSDUH

## Abstract

**Background:**

Despite a number of states in the U.S. enacting medical marijuana policies, there is currently a lack of research outlining the role that individual-level factors play in predicting medical marijuana use, especially regarding use and misuse of prescription pain relievers. The overall aim of this study was to assess the prevalence of medical marijuana use in the U.S. and to identify clinical, social, and demographic predictors.

**Methods:**

A retrospective secondary database analysis was conducted utilizing five years of the National Survey on Drug Use and Health (NSUDH). A multivariable logistic regression model assessed the association between prescription pain reliever use and medical marijuana in the adult U.S. population while adjusting for substance use factors, psychiatric factors, and demographic characteristics.

**Results:**

Within the U.S. adult population from 2015 to 2019, medical marijuana use increased from 1.6% to 2.4%, while appropriate prescription pain reliever use decreased from 33.4% to 27.5%, and prescription pain reliever misuse decreased from 4.7% to 3.7%. Of all marijuana users, 15.1% resided within non-medical marijuana states. Medical marijuana users are more likely to have a serious mental illness (14.0% vs. 4.4%) and a non-marijuana related substance dependence (5.3% vs. 1.2%). Past-year medical marijuana use was significantly more likely to be reported among appropriate users of prescription pain relievers (OR = 1.99, *p* < .001) and misusers (OR = 1.94, p < .001) (relative to nonusers).

**Conclusions:**

Prescription pain reliever appropriate use and misuse were associated with higher odds of medical marijuana use. This study identified a potential treatment gap among individuals residing in states with no medical marijuana availability. These study findings highlight the potential benefits of medical marijuana legalization that future research can build on to guide policy making decisions.

## Highlights

• Medical marijuana use is increasing in the U.S.
• Medical marijuana users were more likely to suffer from a serious mental illness.
• Medical marijuana use is associated with illicit drug use and non-marijuana related substance dependence.
• Prescription pain reliever appropriate users and misusers had twice the odds of medical marijuana use.
• Individuals experiencing pain may be using marijuana to replace opioids.

## 1 Introduction

The prevalence of chronic pain has increased dramatically in the past 20 years and affects a significant proportion of U.S. adults.^1^ In 2016, an estimated 20.4% of U.S. adults (50 million) suffered from chronic pain, defined as experiencing pain on most days or every day in the past six months. Roughly 19.6 million individuals (8.6%) experience high-impact chronic pain, defined as pain that limits life or work activities on most days or every day in the past six months.^2^ Pain has a negative impact on an individual's quality of life and is the most commonly cited condition among Americans receiving disability benefits, with the societal cost of chronic pain estimated to be between $560 to $630 billion per year.^1^

Prescription opioids are a key tool for management of pain, with an estimated 5 to 8 million individuals using opioids for long-term chronic pain management.^1^ Prescription opioids associated with serious adverse events such as physical dependence, addiction, transition to heroin use, overdose, and death.^3^, ^4^, ^5^ The Centers for Disease Control and Prevention (CDC) has issued strict guidelines for opioid prescribing to combat these adverse outcomes and has also shifted focus towards the need to identify other treatment options for chronic pain.^6^

Healthcare professionals have called for the substitution of opioids with medical marijuana for management of chronic pain, given medical marijuana's improved safety profile.^7^, ^8^, ^9^ However, medical marijuana is not a perfect solution for pain management as it has some drawbacks, including cognitive and motor impairment, adverse events, and no standard product formulations.^8^ A meta-analysis of 79 randomized clinical trials tested the benefits and adverse events of cannabinoids in treating a variety of conditions and showed a majority of trials demonstrated improvement in symptoms after cannabinoid use, but statistical significance was not found in all trials. However, cannabinoids were associated with a much greater risk of adverse events such as nausea and disorientation compared to placebo.^10^

Despite the need for further evidence establishing efficacy and safety, medical marijuana for pain management has reached higher levels of public acceptance, as highlighted by the increasing number of states allowing marijuana use for pain treatment. Even with the federal government's prohibition on marijuana use for any purpose, 37 U.S. states and the District of Columbia have legalized medical marijuana as of April 2023.^11^ The passage of such policies in a short period underscores a need for research detailing national rates of medical marijuana use along with information specifying who is engaging in medical marijuana use and what clinical, social, and demographic factors make an individual more likely to use.

Multiple studies have utilized large public national datasets to identify outcomes associated with state medical marijuana policy implementation. Most of these studies had a primary focus on changes in recreational marijuana use prevalence, with mixed findings.^12^, ^13^, ^14^, ^15^, ^16^, ^17^, ^18^, ^19^, ^20^, ^21^ Other secondary outcomes associated with medical marijuana law implementation reported in the above studies included increases in perceived marijuana availability,^13^ lower perception of marijuana use riskiness,^14^ quality of life improvement,^19^, ^20^, ^21^ and higher frequencies of binge drinking among those older than 21.^15^

Previous studies utilizing the National Survey on Drug Use and Health (NSDUH) data have documented the relationships between medical marijuana laws and prescription drug use. Compton et al. examined differences between medical and non-medical marijuana users across all U.S. states utilizing 2013–2014 NSDUH data. They found that medical-marijuana-only users were less likely to misuse prescription stimulants and prescription pain relievers compared to recreational marijuana users.^22^ Wen et al. found no significant relationship between medical marijuana law implementation and non-medical prescription pain reliever use when utilizing 2004–2012 NSDUH data.^15^ However, these findings were contradicted by Caputi and Humphreys, who found that medical marijuana users were significantly more likely to report past-year use of any prescription drugs and past-year misuse of any prescription drugs.^23^ These inconsistent results confirm a gap in the current literature regarding the relationship between medical marijuana use and increase or decrease in prescription drug use and misuse.

Despite the increase in the number of states enacting medical marijuana policies, there is currently a lack of research outlining the role individual-level factors play in predicting medical marijuana use, especially within the context of previous use of prescription opioids. The objectives of the study were: 1) To measure the prevalence of past-year medical marijuana use and the prevalence of past-year prescription pain reliever use among U.S. adults from 2015 to 2019, 2) To identify significant changes in medical marijuana use and prescription pain reliever use from 2015 to 2019, and 3) To evaluate the association between past-year prescription pain reliever use and medical marijuana use. The practical implications of achieving these objectives include helping both healthcare providers and policymakers recognize specific patient characteristics that may be indicative of the need for and use of medical marijuana use. This information can help optimize therapeutic outcomes for patients and identify areas for further investigation.

## 2 Material and methods

### 2.1 Data source and study design

A retrospective secondary database analysis was conducted utilizing five years (2015–2019) of NSDUH data, an annual survey of noninstitutionalized U.S. civilians 12 years or older. This design was chosen to gain insights on a wide range of social and demographic factors over a multiple year time period among a nationally representative sample. The NSDUH provides data on tobacco, alcohol, marijuana, prescription medications (including pain relievers, tranquilizers, stimulants, and sedatives), illicit substances use, and information on mental health and psychiatric services utilization.^24^ NSDUH employs an independent stratified multistage area probability sampling method to produce representative results at national and state levels for all 50 states and the District of Columbia. Each state is stratified into approximately equally populated state sampling regions, and census tracts within each state sampling region are selected, followed by census block groups within census tracts and area segments. Dwelling units are then selected within area segments, and up to two residents over 12 years of age in each dwelling unit are selected for the interview.^25^ Lastly, a professional interviewer conducts the in-person interview at the residence. Participation in NSDUH is voluntary, and the participant receives $30 in cash compensation.^26^ We used de-identified public use data files for this project. These datasets contains no identifiable information. The University of Mississippi Institutional Review Board (IRB) determined that secondary analysis of pre-approved public data files, including the NSDUH, does not constitute human subjects research and therefore does not require IRB approval.

### 2.2 Study sample

The study included respondents from the 2015 to 2019 NSDUH interviews. Despite the addition of a medical marijuana use question to the NSDUH in 2013, significant changes were implemented in the measurement of pain reliever use and medical marijuana use beginning in the 2015 survey, and the U.S. Substance Abuse and Mental Health Services Administration (SAMHSA) states that pre-2015 data should not be compared to post-2015 data.^27^ The NSDUH interviewee population consists of residents of U.S. households, individuals in noninstitutionalized housing and civilians living within U.S. military bases.^28^ Due to the NSDUH methodological design, our study excluded those with no fixed address, active-duty military personnel, and institutionalized civilians residing in prisons, residential substance abuse treatment facilities, nursing homes, mental institutions, and long-term care hospitals. Our study also excluded individuals under 18 because of differences in NSDUH's definitions and operationalization of substance use and mental health disorders in minors versus adults. Individuals with missing responses for medical marijuana use were excluded from the regression analyses. Due to data limitations, participants cannot be tracked longitudinally from year to year, and surveys from different years may contain unidentifiable duplicate respondents.

### 2.3 Study variables

The key outcome variable measured in this study was any medical marijuana use in the past year. If respondents indicated that they had used marijuana in the past year, an additional question is prompted: “Earlier, you reported using marijuana in the past year. Was any of your marijuana use in the past 12 months recommended by a doctor or other health care professional?” Individuals that responded to the question affirmatively were identified as using medical marijuana in the past year. An NSDUH-created variable indicated whether respondents were living in a state where a law allowing marijuana use for medical reasons had been passed at the time of the interview. Individuals were able to answer “yes” to the medical marijuana use question, regardless of whether they reside in a state with medical marijuana policies in place.

The study's primary exposure measure was any past-year prescription pain reliever use. Individuals were split into three exclusive groups for this measure: any past-year prescription pain reliever use but no misuse (defined as appropriate use), any past-year prescription pain reliever misuse, and no past year pain reliever use or misuse. NSDUH defines any use of prescription drugs as “either the use of one's own prescription medication as directed by a doctor or the misuse of prescription drugs.” Prescription drug misuse is defined as “use in any way not directed by a doctor, including use without a prescription of one's own medication; use in greater amounts, more often, or longer than told to take a drug; or use in any other way not directed by a doctor.” SAMHSA cautions researchers against using the lifetime use or misuse of prescription drug variables in analyses, due to potential recall bias.^29^ For this reason, the study only used past 12-month use variables for all analyses.

Demographic covariates included age, gender, race, employment status, health insurance status, population density, self-reported health status, and residing in a medical marijuana state. The psychiatric and substance use covariates were serious mental illness (SMI), major depressive episode (MDE), heavy alcohol use, cocaine use, heroin use, stimulant misuse, nicotine dependence, non-marijuana-related substance use disorder, and perceived risk of smoking marijuana 1 to 2 times per week. These factors were based on a previous study by Compton et al. identifying sociodemographic, mental, and physical health characteristics associated with medical marijuana use utilizing the 2013–2014 NSUDH datasets.^22^ All covariates were measured as past-year prevalence, except for heavy alcohol use and nicotine dependence, which are reported as past-month usage. Wherever directly reported demographic characteristics were unavailable, values logically assigned in the editing process or statistically imputed by SAMHSA were used.

### 2.4 Data analysis

Data from all five years of surveys were combined. Weighted samples adjusting for NSDUH-created sampling weights, clustering, and data stratification were reported. The complex survey design features of the NSDUH data were taken into account during the analysis using the survey analysis procedures in SAS (e.g., PROC SURVEYLOGISTIC). Descriptive statistics depicted baseline individual demographic characteristics, medical marijuana use prevalence, and prescription pain reliever use prevalence. For categorical variables, frequency and percentage distributions were reported. Statistical comparisons were conducted between medical marijuana users and nonusers using Pearson chi-square tests to identify significant differences. Means and standard deviations were reported for continuous variables, and *t*-tests were used to identify significant differences. An analysis of variance (ANOVA) was conducted to determine significant changes in medical marijuana use and prescription pain reliever use across the five years of survey data.

A multivariable logistic regression model was utilized to assess the association between prescription pain reliever use and medical marijuana use while adjusting for the substance use factors, psychiatric factors, and demographic characteristics. Because individuals can respond “yes” to past year medical marijuana use regardless of whether they were living in a medical marijuana approved state, descriptive statistics were run to assess the number of individuals using medical marijuana in states with no medical marijuana policies. An additional multivariable logistic regression model was estimated exclusively among individuals residing in non-medical marijuana states to address gaps in access to medical marijuana. The DOMAIN statement in SAS was used to account for the subsetted data analysis. A new sampling weight was created to allow for analysis of the combined data. Following NSDUH guidelines, the new weight variable was created by dividing the single-year weights by the number of data years used (five) so that the estimated number of individuals reported is representative of the national population.^29^ All results were reported on a per-year basis. Odds ratios with 95% confidence intervals were reported for all variables in the model. All data management and analysis were done using SAS Enterprise Guide version 6.1.

## 3 Results

The final weighted study sample consisted of an average of 246,733,772 individuals per year from 2015 to 2019. Past-year medical marijuana use prevalence was reported in 4,771,828 respondents (1.94%), of which 3,080,831 (1.25%) reported only using medical marijuana in the past year, and 1,682,752 (0.68%) reported using both medical marijuana and recreational marijuana in the past year. As for prescription pain reliever use, 75,151,310 individuals (30.46%) used prescription pain relievers appropriately in the past year, and 10,147,676 individuals (4.11%) reported misusing prescription pain relievers in the past year. Demographic characteristics of the study population across the past-year medical marijuana use groups (use vs. no use) are outlined in Table 1. Individuals who used medical marijuana in the past year were primarily young males belonging to any racial group other than Asian, employed part-time or unemployed, uninsured, living in a large metropolitan area, and with lower self-reported health status.

**Table 1:** Individual demographic characteristics stratified by past-year medical marijuana use.

	Medical Marijuana Use		No Medical Marijuana Use		χ^2^	*p*-value
	n	%	n	%		
Total	4,771,828	1.9%	241,141,839	98.1%		
Age					687.9	<0.001
18–25	972,176	20.4%	33,124,643	13.7%		
26–34	1,180,298	24.7%	38,092,356	15.8%		
35–49	1,219,579	25.6%	59,467,034	24.7%		
50–64	1,038,082	21.8%	61,161,273	25.4%		
65+	361,693	7.6%	49,296,533	20.4%		
Gender						
Male	2,759,781	57.8%	115,845,612	48.0%	156.2	<0.001
Female	2,012,047	42.2%	125,296,228	52.0%		
Race					218.7	<0.001
White	3,083,728	64.6%	154,103,891	63.9%		
African American	534,999	11.2%	28,583,159	11.9%		
Asian	98,852	2.1%	13,677,230	5.7%		
Hispanic	798,288	16.7%	38,591,980	16.0%		
Other	255,961	5.4%	6,185,579	2.6%		
Employment Status					169.4	<0.001
Full-time	2,053,064	43.0%	119,565,848	49.6%		
Part-time	737,805	15.5%	31,427,562	13.0%		
Unemployed	369,462	7.7%	10,239,131	4.2%		
Other	1,611,497	33.8%	79,909,299	33.1%		
Health Insurance Status					35.6	<0.001
Not Insured	605,042	12.7%	23,828,434	9.9%		
Insured	4,166,786	87.3%	217,313,405	90.1%		
Population Density					32.6	0.004
1 million or more	2,709,988	56.8%	130,134,720	54.0%		
Fewer than 1 million	1,874,683	39.3%	96,922,932	40.2%		
Not in a CBSA	187,158	3.9%	14,084,187	5.8%		
Health Status					523.9	<0.001
Excellent	663,465	13.9%	51,089,339	21.2%		
Very Good	1,411,621	29.6%	86,567,510	35.9%		
Good	1,507,646	31.6%	70,453,051	29.2%		
Fair/Poor	1,188,321	24.9%	32,992,918	13.7%		
Missing	775	0.0%	39,021	0.0%		

The distribution of substance use and psychiatric factors across past-year medical marijuana use groups are outlined in Table 2. Individuals who used medical marijuana in the past year were significantly more likely to have a serious mental illness (SMI), past-year major depressive episode, past-month heavy alcohol use (12.0% vs. 6.5%, *p* < .001), past-year cocaine use (10.0% vs. 2.0%, *p* < .001), past-year heroin use (1.4% vs. 0.3%, p < .001), past-year stimulant misuse (4.9% vs. 1.9%, p < .001), past-month nicotine dependence (24.9% vs. 10.8%, p < .001), and past-year non-marijuana related substance dependence (5.3% vs. 1.2%, *p* < .001). Additionally, medical marijuana users were more likely to perceive the risk of smoking marijuana 1 to 2 times a week as no risk to moderate risk compared to high risk (97.8% vs. 66.4%, *p* < .001) and were significantly more likely to reside in a medical marijuana state (84.9% vs. 58.2%, *p* < .001). Lastly, medical marijuana users were significantly more likely to report past-year pain reliever appropriate use (45.2% vs. 30.2%, p < .001) and past-year pain reliever misuse (11.4% vs. 4.0%, p < .001).

**Table 2:** Individual substance use and psychiatric factors stratified by past-year medical marijuana use.

	Medical Marijuana Use		No Medical Marijuana Use		χ^2^	p-value
	n	%	n	%		
Total	4,771,828	1.9%	241,141,839	98.1%		
Serious Mental Illness (SMI)					859.6	<0.001
No SMI	4,103,899	86.0%	230,557,975	95.6%		
SMI	667,929	14.0%	10,583,864	4.4%		
Major Depressive Episode (MDE)					727.1	<0.001
No MDE	3,856,462	80.8%	222,055,438	92.1%		
MDE	840,377	17.6%	16,507,026	6.8%		
Missing	74,989	1.6%	2,579,375	1.1%		
Heavy Alcohol Use					194.7	<0.001
No	4,200,290	88.0%	225,394,700	93.5%		
Yes	571,538	12.0%	15,747,139	6.5%		
Cocaine Use					1276.6	<0.001
No	4,293,552	90.0%	236,417,619	98.0%		
Yes	478,276	10.0%	4,724,220	2.0%		
Heroin Use					129.9	<0.001
No	4,706,617	98.6%	240,364,838	99.7%		
Yes	65,211	1.4%	777,002	0.3%		
Stimulant Misuse					180.3	<0.001
No	4,538,144	95.1%	236,449,043	98.1%		
Yes	233,684	4.9%	4,692,796	1.9%		
Nicotine Dependence					809.7	<0.001
No	3,585,707	75.1%	215,016,584	89.2%		
Yes	1,186,121	24.9%	26,125,255	10.8%		
Non-Marijuana SUD					526.2	<0.001
No	4,518,644	94.7%	238,188,712	98.8%		
Yes	253,184	5.3%	2,953,127	1.2%		
Perceived Risk of Marijuana Use					1736.8	<0.001
No Risk to Moderate Risk	4,665,562	97.8%	160,180,676	66.4%		
Great Risk	93,870	2.0%	76,763,699	31.8%		
Missing	12,396	0.3%	4,197,464	1.7%		
Prescription Pain Reliever Use					1157.0	<0.001
No Use	2,071,699	43.4%	158,827,899	65.9%		
Appropriate Use	2,157,356	45.2%	72,739,012	30.2%		
Misuse	542,773	11.4%	9,574,928	4.0%		
Medical Marijuana State					1197.6	<0.001
No	719,609	15.1%	100,802,805	41.8%		
Yes	4,052,220	84.9%	140,339,034	58.2%		

**Fig. 1:**
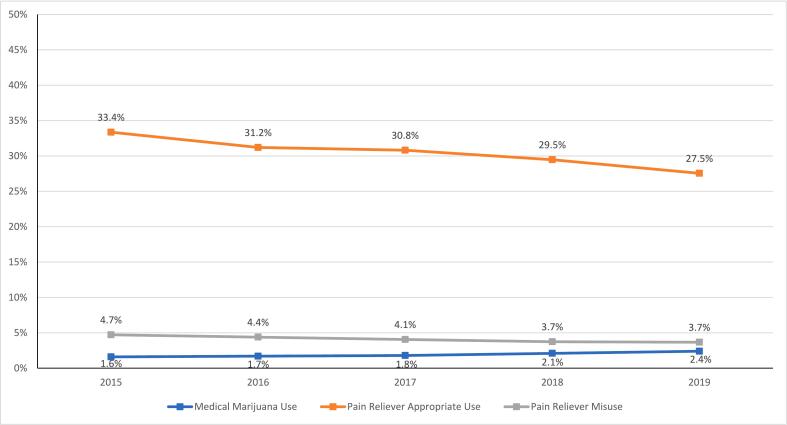
Changes in Prescription Pain Reliever Use, Misuse, and Medical Marijuana Use from 2015 to 2019.

The results of the ANOVA determining significant changes in medical marijuana use and prescription pain reliever use across the five years of survey data are reported in Fig. 1. The results indicate a continually steady and significant increase in medical marijuana use, with prevalence growing from 1.6% in 2015 to 2.4% in 2019 (F = 85.73, *p* < .001). For pain relievers, the results indicated a significant decrease in appropriate pain reliever use, with prevalence declining from 33.4% in 2015 to 27.5% in 2019, along with a significant decrease in pain reliever misuse, with prevalence declining from 4.7% in 2015 to 3.7% in 2019 (F = 478.04, *p* < .001).

The multivariable logistic regression model identified predictive factors of medical marijuana use among all study eligible individuals. Table 3 outlines the results and reports odds ratios for medical marijuana use in the past year for each predictor. When compared to the nonusers, individuals who used pain relievers appropriately as well as those who misused pain relievers had significantly higher odds of using medical marijuana in the past year (appropriate use OR = 1.99, 95% confidence interval [CI] = 1.79–2.22, *p* < .001; misuse OR = 1.94, CI = 1.62–2.33, p < .001), after accounting for all demographic, substance use, and psychiatric risk factors. Demographic characteristics that were found to be significant predictors of greater odds of medical marijuana use included the 26–34 age group, male gender, “other” race, part-time employment and unemployment status, and lower self-reported health status. Demographic characteristics that were found to be significant predictors of lower odds of medical marijuana use included Asian race, and older age groups. Psychiatric and substance use factors that were significant predictors of increased medical marijuana use included SMI, MDE, cocaine use, nicotine dependence, lower perceived risk of marijuana use, and residing in a medical marijuana state. Heroin use was a significant predictor of decreased medical marijuana use.

**Table 3:** Adjusted odds ratios predicting past-year medical marijuana use.

Variable	Odds Ratio	95% LCL	95% UCL	p-value
Prescription Pain Reliever Use (Ref = No Use)				
Appropriate Use	1.99	1.79	2.22	<0.001
Misuse	1.94	1.62	2.33	<0.001
Age (Ref = 18–25)				
26–34	1.13	1.03	1.24	0.011
35–49	0.85	0.76	0.95	0.004
50–64	0.63	0.53	0.74	<0.001
65+	0.31	0.24	0.40	<0.001
Gender (Ref = Female)				
Male	1.45	1.31	1.61	<0.001
Race (Ref = White)				
African American	0.99	0.86	1.13	0.861
Asian	0.50	0.38	0.66	<0.001
Hispanic	1.02	0.90	1.16	0.748
Other	1.43	1.20	1.72	<0.001
Employment Status (Ref = Full-time)				
Part-time	1.46	1.25	1.70	<0.001
Unemployed	1.57	1.31	1.88	<0.001
Other	1.65	1.50	1.82	<0.001
Health Insurance Status (Ref = Insured)				
Not Insured	1.12	0.99	1.27	0.073
Population Density (Ref = Fewer than 1 million)				
1 million or more	1.04	0.95	1.14	0.387
Not in a CBSA	0.83	0.62	1.12	0.221
Self-Reported Health Status (Ref = Excellent)				
Very Good	1.09	0.96	1.25	0.186
Good	1.39	1.19	1.63	<0.001
Fair/Poor	2.15	1.84	2.51	<0.001
Serious Mental Illness (Ref = No SMI)				
SMI	1.40	1.20	1.62	<0.001
Major Depressive Episode (Ref = No MDE)				
MDE	1.31	1.11	1.55	0.002
Heavy Alcohol Use (Ref = No)				
Yes	1.03	0.89	1.19	0.718
Cocaine Use (Ref = No)				
Yes	2.38	2.02	2.81	<0.001
Heroin Use (Ref = No)				
Yes	0.61	0.41	0.91	0.016
Stimulant Misuse (Ref = No)				
Yes	0.84	0.69	1.02	0.083
Nicotine Dependence (Ref = No)				
Yes	1.54	1.38	1.72	<0.001
Non-Marijuana Substance Dependence (Ref = No)				
Yes	1.05	0.85	1.29	0.644
Perceived Risk of Marijuana Use (Ref = Great Risk)				
No Risk to Moderate Risk	15.80	12.28	20.34	<0.001
Residing in Medical Marijuana State (Ref = Nonmedical State)				
Medical Marijuana State	4.25	3.79	4.78	<0.0001

The second multivariable logistic regression model attempted to identify predictive factors of medical marijuana use only among individuals residing in non-medical marijuana states. Table 4 outlines the results and reports the odds ratios for medical marijuana use in the past year strictly among individuals living in states with no medical marijuana policies in place. When compared to the nonusers, individuals engaged in both pain reliever appropriate use and pain reliever misuse had significantly higher odds of past-year medical marijuana use (appropriate use OR = 1.80, CI = 1.41–2.28, *p* < .001; misuse OR = 2.11, CI = 1.49–2.99, p < .001). Demographic characteristics that were significant predictors of increased odds of medical marijuana use among non-medical marijuana state residents included male gender, unemployment, and lower self-reported health status. Demographic characteristics that were significant predictors of lower odds of medical marijuana use included older age groups and Asian race. Psychiatric and substance use factors that were significant predictors of increased medical marijuana use among non-medical marijuana state residents included cocaine use, nicotine dependence, and lower perceived risk of marijuana use.

**Table 4:** Adjusted odds ratios predicting past-year medical marijuana use (non-medical marijuana state residents only).

Variable	Odds Ratio	95% LCL	95% UCL	p-value
Pain Reliever Use (Ref = No Use)				
Appropriate Use	1.80	1.41	2.28	<0.001
Misuse	2.11	1.49	2.99	<0.001
Age (Ref = 18–25)				
26–34	0.86	0.72	1.03	0.090
35–49	0.59	0.46	0.74	<0.001
50–64	0.33	0.23	0.47	<0.001
65+	0.10	0.06	0.19	<0.001
Gender (Ref = Female)				
Male	1.47	1.17	1.84	0.002
Race (Ref = White)				
African American	1.20	0.96	1.48	0.102
Asian	0.24	0.09	0.62	0.004
Hispanic	0.81	0.54	1.20	0.286
Other	1.30	0.90	1.88	0.153
Employment Status (Ref = Full-time)				
Part-time	1.16	0.84	1.60	0.358
Unemployed	2.07	1.46	2.93	<0.001
Other	1.88	1.51	2.33	<0.001
Health Insurance Status (Ref = Insured)				
Not Insured	1.04	0.80	1.35	0.745
Population Density (Ref = Fewer than 1 million)				
1 million or more	1.21	0.97	1.49	0.087
Not in a CBSA	0.80	0.56	1.14	0.213
Self-Reported Health Status (Ref = Excellent)				
Very Good	0.84	0.61	1.17	0.303
Good	1.20	0.89	1.61	0.225
Fair/Poor	2.21	1.63	2.99	<0.001
Serious Mental Illness (Ref = No SMI)				
SMI	1.60	0.98	2.59	0.059
Major Depressive Episode (Ref = No MDE)				
MDE	1.12	0.70	1.78	0.636
Heavy Alcohol Use (Ref = No)				
Yes	0.92	0.70	1.21	0.535
Cocaine Use (Ref = No)				
Yes	2.25	1.53	3.31	<0.001
Heroin Use (Ref = No)				
Yes	1.32	0.68	2.60	0.406
Stimulant Misuse (Ref = No)				
Yes	0.99	0.69	1.42	0.951
Nicotine Dependence (Ref = No)				
Yes	2.25	1.75	2.89	<0.001
Non-Marijuana Substance Dependence (Ref = No)				
Yes	1.00	0.69	1.43	0.986
Perceived Risk of Marijuana Use (Ref = Great Risk)				
No Risk to Moderate Risk	7.96	4.63	13.69	<0.001

## 4 Discussion

This study builds off previous literature^17^^,^^18^ and examines the association between medical marijuana use and prescription pain reliever use and misuse utilizing a nationally-representative dataset. This study found that roughly 1.9% of the U.S. population engaged in medical marijuana use every year between 2015 and 2019. Medical marijuana users were primarily young males belonging to any racial group other than Asian, living in urban areas who are not employed full-time and are self-perceived as less healthy. During the same period, roughly 30.5% of the U.S. population used prescription pain relievers appropriately, and 4.1% misused pain relievers in a way not prescribed by a doctor. When observing time trends in prevalence, we found that medical marijuana use steadily increased every year from 2015 to 2019, while prescription pain reliever appropriate use and misuse steadily decreased yearly. These findings reflect current trends in the U.S., such as increases in the acceptance of marijuana legalization^30^ and stricter federal and state laws surrounding opioid prescribing.^31^

This study's central finding was that prescription pain reliever appropriate users and misusers had nearly twice the odds of past-year medical marijuana use compared to individuals who did not use pain relievers in the past year. This finding may indicate that pain patients receiving opioids or individuals in need of pain relief may be using marijuana to replace opioids. However, due to the cross-sectional nature of NSDUH data, we could not establish the temporal relationship between individuals' marijuana use and pain reliever use. However, this conclusion is supported by previous research. For example, Ishida et al. found that 41% of individuals who use both marijuana and opioids in the past year reported a decrease or cessation of opioid use due to their marijuana use, and the reported reasons for decreased use were primarily valid medical reasons (better pain management, fewer side effects, no withdrawal symptoms, etc.).^32^ These study findings highlight the potential benefits of medical marijuana legalization while also uncovering a need for prospective future research that establishes the causal relationship between medical marijuana use and prescription pain reliever use.

Demographic, psychiatric, and substance use factors that predict medical marijuana use were also identified. Our study results agree and disagree with the inconsistent findings in previous literature. Consistent with previous literature, there were higher odds of medical marijuana use among younger individuals, males, those with lower self-reported health, and those who view marijuana use as less risky.^16^^,^^22^^,^^33^ Alternatively, results indicated no significant correlation between medical marijuana use and heavy alcohol use or prescription stimulant misuse, which contradicts previous study results.^22^^,^^23^ These are important findings as they provide an in-depth and more recent look at the population engaging in medical marijuana use along with reporting potentially significant changes in the patient population.

A significant secondary finding of this study is the high prevalence of serious mental illness (SMI), and major depressive episodes (MDE) reported among medical marijuana users. NSDUH defines an SMI as a mental, behavioral, or emotional disorder that results in serious functional impairment and interferes with major life activities, while MDE is defined as experiencing a depressed mood for at least two weeks in the past year along with a having a majority of specified depression symptoms.^34^ Individuals with a past-year SMI had 1.4 times the odds of medical marijuana use, and individuals with a past-year MDE had 1.3 times the odds of use. These results suggest that healthcare professionals may be prescribing medical marijuana to patients suffering from severe mental illnesses despite the lack of current evidence for its effectiveness in treating psychiatric disorders.^10^ However, we are unable to determine the reason why patients are using medical marijuana due to data limitations. Therefore, this conclusion may need to be interpreted with caution, since mental illness may be a comorbidity as opposed to the indication for medical marijuana use.

The high prevalence of medical marijuana use among residents of non-medical marijuana states was another notable finding of this study. Of all individuals who reported past-year medical marijuana use, 15.1% resided within a state where medical marijuana was not legally available. There are two potential explanations for this high prevalence. First, it is possible that medical marijuana patients living close to state lines of a medical marijuana state may be crossing the state border to be medically recommended and receive medical marijuana. Second, these findings may suggest that medical professionals might be recommending medical marijuana use to patients regardless of legalization status, as concluded by Compton et al.^22^^,^^35^

Further examining this population of individuals living in states without legal marijuana legislation can highlight the tendencies which lead to seeking out medical marijuana despite greater barriers. Given the high percentage of medical marijuana use in non-medical marijuana states, there is clearly an unmet need among this population. These results imply that there could be a treatment gap for potential medical marijuana patients who lack access to treatment. Individuals with poor health or those having severe psychiatric conditions living in non-medical marijuana states could benefit from implementing such policies. For this reason, medical marijuana policies should be expanded to reach these residents and close the potential treatment gap. Uncovering structural barriers, including geographic location, in the path to obtaining medical marijuana establishes a foundation for forthcoming policies and initiatives. Implementing evidence-based strategies and programs to enhance medical marijuana accessibility among prescription pain reliever patients can reduce the inequality in access experienced by eligible patients.

Despite its strengths, the limitations of this study should be acknowledged. The study's primary limitation was the cross-sectional nature of NSDUH data, which limits drawing casual inferences or temporality among study variables. The impact of this limitation was reduced through the use of multiple years of surveys. The results of this study may not be generalizable to the institutionalized populations because they are not sampled in the NSDUH data. These may be important populations to examine in future research due to the likelihood of these individuals experiencing chronic pain. The study also excluded children from the analysis due to differences in how NSDUH operationalizes certain variables in adults versus children. NSDUH also does not include information for pain-related diagnoses, which could be an essential factor in the relationship between medical marijuana use and prescription pain reliever use. Lastly, while it is not expected to bias the results, self-reported data may be subject to recall bias and social desirability bias, which may lead to the under-reporting of drug use prevalence.

The results of this study have several implications for policymakers, healthcare providers, and researchers. First, this study adds to the current body of literature surrounding the medical marijuana patient population by providing a more comprehensive and up-to-date description of individuals engaging in medical marijuana use. Second, the study identified a wide range of individual-level demographic, psychiatric, and substance use factors associated with medical marijuana use, which future studies can build upon to identify changes and trends within the medical marijuana patient population. Lastly, the study results can help policymakers better understand the population that engages in medical marijuana use to help prepare for future medical marijuana policy implementation.

## 5 Conclusion

The use of prescription pain relievers, both appropriately and inappropriately, is decreasing among the U.S. adult population. On the other hand, the use of medical marijuana is increasing. This study concluded that prescription pain reliever appropriate use and misuse was associated with significantly higher odds of medical marijuana use compared to nonusers. Future research must examine this relationship in a more in-depth manner using a longitudinal study designed to provide evidence for how patients could benefit from the expansion of medical marijuana policies.

## Ethics approval

The University of Mississippi Institutional Review Board (IRB) determined that secondary analysis of pre-approved public data files, including the NSDUH, does not constitute human subjects research and therefore does not require IRB approval.

## Funding sources

This research received no specific grant from any funding agency in the public, commercial, or not-for-profit sectors.

## Declaration of Competing Interest

The authors declare that they have no known competing financial interests or personal relationships that could have appeared to influence the work reported in this paper.

## References

[1] Reuben D.B., Alvanzo A.A.H., Ashikaga T. (2015). National institutes of health pathways to prevention workshop: the role of opioids in the treatment of chronic pain. Ann Intern Med.

[2] Dahlhamer J., Lucas J., Zelaya C. (2018). Prevalence of chronic pain and high-impact chronic pain among adults — United States, 2016. Morb Mortal Wkly Rep.

[3] Okie S. (2010). A flood of opioids, a rising tide of deaths. N Engl J Med.

[4] Chou R., Turner J.A., Devine E.B. (2015). The effectiveness and risks of long-term opioid therapy for chronic pain: a systematic review for a national institutes of health pathways to prevention workshop. Ann Intern Med.

[5] Volkow N.D., McLellan A.T. (2016). Opioid abuse in chronic pain--misconceptions and mitigation strategies. N Engl J Med.

[6] Dowell D. (2022). CDC clinical practice guideline for prescribing opioids for pain — United States, 2022. MMWR Recomm Rep.

[7] Choo E.K., Feldstein Ewing S.W., Lovejoy T.I. (2016). Opioids out, cannabis in negotiating the unknowns in patient care for chronic pain. JAMA..

[8] Hasin D.S. (2018). US epidemiology of cannabis use and associated problems. Neuropsychopharmacol..

[9] Patel A., Fee D., Brust J., Song S., Miller T. (2014).

[10] Whiting P.F., Wolff R.F., Deshpande S. (2015). Cannabinoids for medical use: a systematic review and meta-analysis. JAMA..

[11] ProCon.org (2023). Legal Medical Marijuana States and DC. Medical Marijuana. https://medicalmarijuana.procon.org/legal-medical-marijuana-states-and-dc/.

[12] Cerdá M., Wall M., Keyes K.M., Galea S., Hasin D. (2012). Medical marijuana laws in 50 states: investigating the relationship between state legalization of medical marijuana and marijuana use, abuse and dependence. Drug Alcohol Depend.

[13] Martins S.S., Mauro C.M., Santaella-Tenorio J. (2016). State-level medical marijuana laws, marijuana use and perceived availability of marijuana among the general U.S. population. Drug Alcohol Depend.

[14] Wall M.M., Poh E., Cerdá M., Keyes K.M., Galea S., Hasin D.S. (2011). Adolescent marijuana use from 2002 to 2008: higher in states with medical marijuana laws, cause still unclear. Ann Epidemiol.

[15] Wen H., Hockenberry J.M., Cummings J.R. (2015). The effect of medical marijuana laws on adolescent and adult use of marijuana, alcohol, and other substances. J Health Econ.

[16] Harper S., Strumpf E.C., Kaufman J.S. (2012). Do medical marijuana laws increase marijuana use? Replication study and extension. Ann Epidemiol.

[17] Garg R., Shojania K., De Vera M.A. (2022). The association between cannabis and codeine use: a nationally representative cross-sectional study in Canada. J Cannabis Res.

[18] Ramadan M.M., Banta J.E., Bahjri K., Montgomery S.B. (2021). Marijuana users are likely to report opioid misuse among adults over 50 years in representative sample of the United States (2002-2014). J Addict Dis.

[19] Greis A., Larsen E., Liu C., Renslo B., Radakrishnan A., Wilson-Poe A.R. (2022). Perceived efficacy, reduced prescription drug use, and minimal side effects of cannabis in patients with chronic orthopedic pain. Cannabis Cannabinoid Res.

[20] Aviram J., Lewitus G.M., Vysotski Y. (2021). Prolonged medical cannabis treatment is associated with quality of life improvement and reduction of analgesic medication consumption in chronic pain patients. Front Pharmacol.

[21] Lucas P., Boyd S., Milloy M.J., Walsh Z. (2021). Cannabis significantly reduces the use of prescription opioids and improves quality of life in authorized patients: results of a large prospective study. Pain Med.

[22] Compton W.M., Han B., Hughes A., Jones C.M., Blanco C. (2017). Use of marijuana for medical purposes among adults in the United States. JAMA..

[23] Caputi T.L., Humphreys K. (2018). Medical marijuana users are more likely to use prescription drugs medically and nonmedically. J Addict Med.

[24] Substance Abuse and Mental Health Services Administration (2023). NSDUH - About the Survey. https://nsduhweb.rti.org/respweb/about_nsduh.html.

[25] Substance Abuse and Mental Health Services Administration (2023). Section 2: Sample Design Report. https://www.samhsa.gov/data/sites/default/files/cbhsq-reports/NSDUHmrbSampleDesign2018/NSDUHmrbSampleDesign2018.pdf.

[26] Substance Abuse and Mental Health Services Administration (2023). National Survey on Drug Use and Health | CBHSQ Data. https://www.samhsa.gov/data/data-we-collect/nsduh-national-survey-drug-use-and-health.

[27] Substance Abuse and Mental Health Services Administration (2015). https://www.samhsa.gov/data/sites/default/files/NSDUH-RedesignChanges-2015.pdf.

[28] Substance Abuse and Mental Health Services Administration (2019). https://www.samhsa.gov/data/sites/default/files/cbhsq-reports/NSDUHMethodsSummDefs2018/NSDUHMethodsSummDefs2018.htm.

[29] Center for Behavioral Health Statistics and Quality (2023). 2018 National Survey on Drug Use and Health: Final Analytic File Codebook. Substance Abuse and Mental Health Services Administration. https://www.cdc.gov/rdc/data/b1/2018-FinalAnalytic-Codebook-RDC508.pdf.

[30] Green T.V. (2023). https://www.pewresearch.org/fact-tank/2021/04/16/americans-overwhelmingly-say-marijuana-should-be-legal-for-recreational-or-medical-use/.

[31] National Conference of State Legislatures (2023). Prescribing Policies: States Confront Opioid Overdose Epidemic. https://www.ncsl.org/research/health/prescribing-policies-states-confront-opioid-overdose-epidemic.aspx.

[32] Ishida J.H., Wong P.O., Cohen B.E., Vali M., Steigerwald S., Keyhani S. (2019). Substitution of marijuana for opioids in a national survey of US adults. PloS One.

[33] Pedersen E.R., Tucker J.S., Seelam R., Rodriguez A., D’Amico E.J. (2019). Factors associated with acquiring a medical marijuana card: a longitudinal examination of young adults in California. J Stud Alcohol Drugs.

[34] Substance Abuse and Mental Health Services Administration (2023). The NSDUH Report: Revised Estimates of Mental Illness from the National Survey on Drug Use and Health. https://www.samhsa.gov/data/sites/default/files/NSDUH148/NSDUH148/sr148-mental-illness-estimates.htm.

[35] Chaudhry H.J., Hengerer A.S., Snyder G.B. (2016). Medical board expectations for physicians recommending marijuana. JAMA..

